# Research: Is resection of tumours involving the pelvic ring justified? : A review of 49 consecutive cases

**DOI:** 10.1186/1477-7800-2-9

**Published:** 2005-04-09

**Authors:** Alex Yuen, Eugene T Ek, Peter FM Choong

**Affiliations:** 1 Department of Orthopaedics, University of Melbourne, St. Vincent's Hospital, 41 Victoria Parade, Fitzroy, 3065, Victoria, Australia; 2 Sarcoma Unit, Division of Surgical Oncology, Peter MacCallum Cancer Institute, Melbourne, Australia; 3 Department of Surgery, St. Vincent's Hospital, 41 Victoria Parade, Fitzroy, 3065, Victoria, Australia

## Abstract

**Introduction:**

Pelvic surgery is challenging and impacts significantly on limb and visceral function, thus, raising the question "is heroic surgery justifiable". This study assessed the functional, oncologic and surgical outcomes following pelvis tumour resections.

**Methods:**

Between 1996–2003, 49 patients (mean age 43 years) underwent pelvic tumour resections- 38 primary malignant tumours, 5 secondary tumours and 6 benign tumours. Bone tumours comprised 5 osteosarcomas, 5 Ewings sarcomas, and 12 chondrosarcomas. Of the soft tumours, 9 were of neural origin. Tumours involved the ilium, acetabulum, pubic bones, sacrum or a combination of these. Functional assessment was performed and no patient had metastases at presentation.

**Results:**

There were 41 limb sparing resections and 8 hindquarter amputations. Surgical margins were intralesional (1), marginal (13), wide (26), and radical (3). Of limb sparing surgery, prosthetic reconstructions were performed in 10 patients, biologic reconstructions in 6, a combination of these in 3 and no reconstruction in others. There was 1 intraoperative death, 7 local recurrences and 19 metastases. Death from disease occurred at a mean of 14.2 months with a mean followup of 27 (1–96) months. Amputation and periacetabular resections had worse functional outcomes. Emotional acceptance was surprisingly high.

**Conclusion:**

Pelvic resections are complex. Functional outcome is significantly affected by surgery. Disease control is similar to limb tumours. Emotional acceptance of surgery in survivors was surprisingly high. Major pelvic resection for malignancy appears justified.

## Background

Pelvic resection is challenging and can have significant functional, social and psychological impact on the patient. It is also associated great morbidity especially when extensive resection is required to attain adequate surgical margins. With the introduction of adjuvant chemotherapy and radiotherapy, and improved reconstruction techniques, limb- sparing surgery has largely replaced external hemipelvectomy. However, given the often poor prognosis of patients with pelvic tumours, the question of whether this type of surgery is justified remains unanswered. This study assessed the functional, oncologic and surgical outcomes following pelvic tumour resection.

## Methods

### Patients

From 1996 to 2003, 49 patients at St. Vincent's Hospital underwent pelvic tumour resections. Clinical and functional information on all 49 patients were available for review. There were 25 males and 24 females. The mean age was 43.2 (15 – 72) years. 38 patients underwent surgery for primary malignancy, 6 patients for benign tumour, while 5 patients had surgery for metastasis.

### Tumour characteristics

Of the 38 primary malignancy cases, there were 5 osteosarcomas, 5 Ewing sarcoma, and 12 chondrosarcoma. Of the 5 metastatic tumours, there was 1 colorectal cancer, 1 vulvar cancer, 1 renal cell cancer, 1 squamous cell cancer and one Lung cancer. The histological subgroup is summarised in Table 1.

**Table 1:** Tumour Types

*Primary Tumours*	*Number*
Central osteosarcoma	1
Chondroblastic osteosarcoma	4
Paget's Osteosarcoma	1
Undifferentiated sarcoma	1
Ewing Sarcoma	5
Chondrosarcoma	12
Chordoma	4
MFH	4
Desmoid tumour	1
Leiomyosarcoma	1
Malignant periphereal nerve sheath tumour	1
Spindle cell carcinoma	1
Neurofibrosarcoma	1
Rhabdomyosarcoma	1
Neurofibroma	1
Osteochondroma	2
Schwannoma	2
Chondromyxoid fibroma	1
	
*Secondary Tumours*	
Colorectal	1
Vulvar	1
Renal cell cancer	1
Squamous cell carcinoma	1
neuroendocrine cancer from the lung	1

According to the Enneking's classification, one patient had stage 1A tumour, one patient had stage 1B tumour, 6 patients had stage 2A tumours and 30 patients had 2B tumours.

Tumours involved the ilium (P1), the periacetabular region (P2) with or without involvement of the proximal femur (H1), the pubis (P3), the sacrum (P4) or a combination of these. The location and the corresponding histological subgroup is summarised in Table 2.

**Table 2:** Location in relation to type of tumour

	P1	P1/2	P2	P2/H1	P2/3	P3	P4	P1/4
BENIGN	3	0	0	0	0	0	3	0
SARCOMA	5	4	4	4	9	2	5	5
CARCINOMA	1	0	1	0	1	2	0	0

### Operations

**Figure 1:**
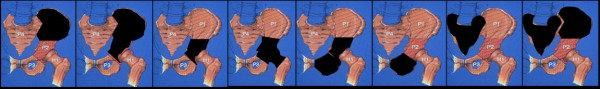
An illustration of the types of pelvic resection.

Pelvic resections were classified according to Enneking and Dunham into 4 types; namely, iliac (T1), acetabular (T2), pubis or ischium (T3) and sacral (T4). Combinations of these resections with or without high femoral resection (H1) were also performed (Figure 1).

The types of resection and the methods of reconstruction are summarised in Table 3.

**Table 3:** Types of resection and Methods of reconstruction

	T1	T1/2	T2	T2/H1	T2/3	T3	T4	T1/4
Limb Sparing Surgery (41)	7	3	0	3	9	3	7	7
Amputation (8)					8			
Reconstruction (19)	1 FVFG 1 pelvic reconstruction with bone cement	3 ischiofemoral pseudoarthrodesis		2 pelvic allograft with hip arthroplasty 1 saddle prosthesis with reconstruction plate	8 saddle prosthesis 1 pelvic allograft with hip arthroplasty			1 FVFG 1 ischiofemoral pseudoarthrodesis

The surgical margins achieved were classified according to the criteria established by the Musculoskeletal Tumour society; namely intralesional, marginal, wide, and radical.

### Adjuvant Treatment

9 patients received chemotherapy alone, 6 received radiotherapy alone, and 6 patients received both chemotherapy and radiotherapy.

### Follow-up

**Figure 2:**
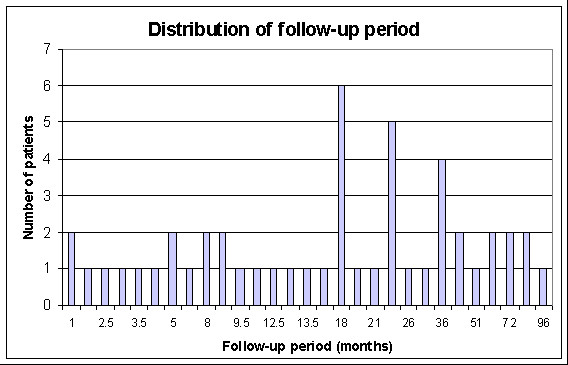
A bar graph showing the distribution of the follow-up periods.

Follow – up was calculated from the time of surgery to the last date of review or death. The series was updated by reviewing the clinical charts of the patients. In addition, a standardized questionnaire regarding clinical outcome and function was completed for every patient by way of phone interview. Evaluation of disease status and functional result at last follow-up review was performed for all patients. The mean length of follow-up was 27 months (1 to 96 months). The distribution of the follow-up period is summarised in Figure 2.

### Functional assessment

Function was assessed by the modified function evaluation system recommended by Enneking et al [1] With this system, functional assessment is based on an analysis of factors (pain, functional activities, emotional acceptance) pertinent to the patient as a whole and factors specific to the lower limb (use of external supports, walking ability, and gait). For each of the six factors, values of 0 to 5 are assigned on the basis of established criteria. Descriptive terms like excellent, good, fair or poor are assigned to a specific numerical range. (26–30 Excellent, 21–25 Good, 16–20 Satisfactory, 11–15 Fair, and 10 or less Poor)

## Results

### Duration of surgery

The mean operative time was 5.2 (1.5 – 10) hours.

### Blood loss

The mean number of packed cell units for intra-operative transfusion was 10 (2 – 26) units.

### Length of stay

The mean length of in – hospital stay was 23 (2 – 110) days.

### Oncologic Outcome

Of the 5 patients who underwent surgery for metastatic carcinoma, marginal margins were achieved in 3 patients, and wide margins were achieved in the other 2 patients.

Of the 38 patients who underwent surgery for sarcoma, intralesional margin was achieved in 1 patient, marginal margins were achieved in 10 patients, wide margins were achieved in 24 patients and radical margins were achieved in 3 patients.

The oncologic outcomes in relation to tumour type, previous biopsy and surgical margin are shown in Table 4. 7 patients had local recurrence. 19 patients developed metastasis. 19 patients died of disease. The mean survival of these patients was 14.2 (1 – 51) months.

**Table 4:** Oncologic outcome in relation to surgical margin, tumour type and previous biopsy.

***MARGINS***				
**CARCINOMA**	**n**	**L.R.**	**Prev. Biopsy**	**Mets**

INTRALESIONAL	0	0	0	0
MARGINAL	3	1	1	1
WIDE	2	0	0	2
**SARCOMA**				
INTRALESIONAL	1	1	1	1
MARGINAL	10	3	1	3
WIDE	24	2	1	9
RADICAL	3	0	0	3

### Complications

22 of the 49 patients had complications.

There was one intraoperative death.

3 patients had common peroneal nerve palsy and 1 patient had sciatic nerve palsy. 2 patients had urinary incontinence and 1 patient had erectile dysfunction after Type 4 pelvic resection.

14 patients had wound infections: 2 of these were superficial infections and 12 were deep infections.

1 patient had a wound hematoma.

3 patients had dislocation/disarticulation of the saddle prosthesis.

3 patients after external hemipelvectomy had phantom limb pain.

13 patients required additional surgeries. 6 of these patients required open drainage or debridement of infected wound. 1 patient required removal of prosthesis due to infection. 2 patients required subsequent split-skin graft. 2 patients required subsequent rectus flaps. 1 patient required a rhomboid flap. 1 patient required a Latissimus Dorsi flap but due to recurrent wound infections subsequently underwent external hemipelvectomy.

The complications in relation to the type of pelvic resection are shown in Table 5.

**Table 5:** Types of resection and complications

	T1	T1/2	T2	T2/H1	T2/3	T3	T4	T1/4	AMP	Total
Infection	2				3	1	1	2	5	14
Hematoma									1	1
skin/flap problem									2	2
Venous thrombus	1	1								2
nerve injury		1		1	2			1		5
Phantom limb pain									3	3
Urinary incontinence							2			2
Erectile dysfunction							1			1
Prosthetic dislocation					3					3
Require additional surgery	1				3	1	2	2	4	13

### Functional Outcome

The outcomes for 44 patients were assessed. According to the criteria of Enneking et al [1], 11 patients had excellent functional results, 7 patients had good functional results, 9 patients had satisfactory functional results, 5 patients had fair functional results, and 12 patients had poor results.

The functional results in relation to the performed surgical procedure are shown in Table 6.

**Table 6:** Functional Result in relation to surgical of procedure

	T1	T1/2	T2	T2/H1	T2/3	T3	T4	T1/4	AMP
Mean Functional Score	22.8	19.3		18	12	27	25	19	5
Range	(12 – 29)	(19 – 20)		(15 – 21)	(3 – 18)		(15 – 30)	(8 – 28)	(0 – 10)
Emotional Acceptance	4.5	4.3		4.5	2.7	4.5	3.9	3.3	2
Functional score excluding the emotional acceptance component	18.3 (73.2%)	15 (60%)		13.5 (54%)	9.3 (37.2%)	22.5 (90%)	21.1 (84.4%)	15.7 (62.8%)	3 (12%)

Of the internal hemipelvectomy, Type 3 and Type 4 pelvic resections have the best mean overall functional scores and are both in the range of excellent functional result. On the other hand, combined type 2 and 3 resection has the worst mean overall functional score and falls in the range of fair functional result.

The mean overall function score for external hemipelvectomy falls in the range of poor functional result.

When the emotional acceptance score is excluded from the overall functional score, Type 3 and Type 4 pelvic resections still have the best total score. Resections involving the hip joint (i.e. T1/2, T2/H1, and T2/3) all scored below 60% of the total.

All internal hemipelvectomy have mean emotional acceptance score of greater than 4 except combined type 2 and 3 pelvic resection, combined type 1 and 4 resection, and type 4 resection. (They scored 2.7, 3.3 and 3.9 respectively)

External hemipelvectomy has the lowest mean emotional acceptance score.

## Discussion

Pelvic resections are complex. They are technically difficult due to the usually large size of pelvic tumours, and the close proximity of pelvic viscera and neurovasculature of the pelvis and lower limb. The technical and human resources required are intensive. Patients often require large amount of blood products. They require post-operative recovery in ICU and in most cases long periods of in-patient rehabilitation. Most importantly, the diagnosis and management of pelvic tumour require a multidisciplinary approach, which includes radiologist, oncologist, physiotherapist, Occupation therapist, and Orthotist. Hence, patients must be treated in specialised centres where such resources are available.

As shown in our series, the morbidity is great with a high risk of wound infection and nerve injury. The overall complication rate has been reported as high as 50–60% [2]. In the current series, 22 out of 49 patients (45%) had complications. Regardless of the type of resection, there is a significant functional, social and psychological impact on the patient. Moreover, there is a significant risk of peri -operative mortality.

Because of these issues, there remains the question as to whether pelvic surgery performed with curative intent or for palliation is justified particularly for patients with high grade malignancy where the prognosis is poor. However, the results of the current series show that this type operation may be worthwhile.

In regards to oncologic outcome, forty out of the forty-nine patients survived longer than six months. The mean survival was greater than one year. The local failure rate was 16%, and 40% of patients developed metastasis. These figures are supported by Pring et al [3] who reported a similar local failure rate of 19% and an overall survival rate of 69%. Ozaki et al [4] reported a higher local recurrence rate of 60% and a poorer 5-year survival rate of 27%. However, in that study only patients with osteosarcoma were studied and 70% of patients had inadequate surgical margin. Disease control by pelvic resection is comparable to that of surgery for limb tumors. Bacci et al [5] reported, in his series of 526 patients with osteosarcoma of the extremities, a local recurrence rate of 6% and an overall survival rate of 70%. Sluga M et al [6] in his series of 130 patients also found a comparable LR rate of 2.3% and OS of 71% for limb-sparing surgery. The comparable oncologic result of pelvic resection to surgery for limb tumours is surprising because the response of pelvic tumors to adjuvant therapy is generally poor and the adequacy of surgical margin difficult to achieve.

The functional outcome varied with the level of resection but more than 50% of patients had satisfactory or better overall functional results in the current series. This is supported by Wirbel et al [7] who also found more than 60% of patients had good or excellent functional results in his series of 93 patients.

In the current study, we observed, not surprisingly, a significantly worse functional score in patients who had hindquarter amputation compared to those who had limb sparing surgery. Emotional acceptance was likewise poor in the hemipelvectomy group. Other series [7,8] have observed similar results. We found that the reasons for the poor emotional acceptance are largely due to loss of mobility and the common complication of phantom limb pain.

In addition, patients who underwent external hemipelvectomy in the current series had the highest rate of wound infection at 56%. They also had the worst survival rate with only 11% of patients alive at the time of follow-up. These figures are supported by Masterson et al [9] who reported a 79% incidence of wound infection and 8 deaths within a year among 22 patients. These results further support the view that careful patient selection is required for hindquarter amputation. In recent years, hemipelvectomy is only performed for patients in whom extensive bone or soft tissue resection makes reconstruction difficult or leaves the leg with poor function. Involvement of the sciatic nerve necessitating resection of the nerve has not been considered a contraindication to limb-sparing surgery [10].

With Limb-Sparing Surgery, Type 3 and Type 4 resections had the best results when the function is examined separately from the emotional acceptance component. On the other hand, resections that involve the hip joint confer the worst results. The reason could be the many problems associated the methods of reconstruction.

Ischiofemoral arthrodesis and pseudoarthrosis are associated with shortening of the leg and lack of mobility [11]. The restricted range of flexion and extension permitted at the pubic symphysis may also result in aching symptoms. They also have long consolidation times, which means patients require longer periods of rehabilitation and use of gait support. Enneking [10] and Menendez [12] found that the maximum possible activity was achieved after an average rehabilitation period of 14.2 months.

Saddle prosthesis is suited for bridging large area of defect in cases where part of the iliac crest could be preserved. This form of endoprosthetic replacement provides good cosmetic result [11]. However, the eccentric position of the new hip center reduces the range of movement. Moreover, if major parts of the ilium were resected, loosening of the prosthesis with lateral shift of the prosthesis could be a long-term problem [7]. In this regard, Dacron ties have been advocated for use to secure the saddle while a pseudo – capsule develops [13]. In the current series, 3 out of 9 patients had dislocation of the prosthesis.

There are other alternatives, which include reconstruction with allografts with or without hip arthroplasty. This was performed for 3 patients in the current series. Their overall functional result was only fair to satisfactory but all had high emotional acceptance score. Langlais et al [14] found, in a small series of 12 patients, 8 patients had good to excellent overall functional results. Bell at al [15] in a larger series of 17 patients similarly found a high functional result among his patients. However, there are major complications associated with this procedure in particular deep infection and graft disintegration. The risks of these complications are relatively high [16]. Moreover, this procedure is restricted by the availability of allograft bone [17].

Regardless of the functional outcome, it is interesting that most patients except those who had amputation found the procedure acceptable. This is supported by Hillmann et al [2] who also found high acceptance scores for all types of resection except amputation and acetabular resection with pelvic prosthetic reconstruction. The high emotional acceptance of patients is probably the most important factor that advocates pelvic resections: particularly limb sparing surgery.

It should be noted that the recent introduction of transxemic acid has had a significant impact in the reduction of operative bleeding and need for blood transfusion. Transxemic acid has been shown to be effective in many types of surgery including major cardiac surgery, liver transplantation, and hip and knee arthroplasty [18-21]. Although not examined in the current study, its use in pelvic surgery will likely to have the same beneficial effect.

## Conclusion

Pelvic surgery is challenging. The morbidity may be great and requirements on technical and human resources are high. Disease control is similar to limb tumours. Functional result and emotional acceptance of patients are generally high. The relief of pain and improvement in function in both curative and palliative setting is extremely rewarding. Major pelvic resection for malignancy appears justified.

## Competing Interests

The author(s) declare that they have no competing interests.

## Authors' Contributions

Dr. Alex Yuen collected the clinical data, wrote and prepared manuscript.

Dr. Eugene Ek participated in data collection.

Prof Peter Choong carried out the surgery, and participated in data collection and preparation of manuscript.
